# Biotic interactions, evolutionary forces and the pan-plant specialized metabolism

**DOI:** 10.1098/rstb.2023.0362

**Published:** 2024-09-30

**Authors:** Sophie de Vries, Ivo Feussner

**Affiliations:** ^1^ Department of Applied Bioinformatics, Institute for Microbiology and Genetics, University of Goettingen, Goldschmidtstr. 1, Goettingen 37077, Germany; ^2^ Department of Plant Biochemistry, Albrecht-von-Haller-Institute for Plant Sciences and Goettingen Center for Molecular Biosciences (GZMB), University of Goettingen, Justus-von-Liebig Weg 11, Goettingen 37077, Germany

**Keywords:** plant evolution, plant–microbe interaction, specialized metabolism, non-model organisms, streptophytes

## Abstract

Plant specialized metabolism has a complex evolutionary history. Some aspects are conserved across the green lineage, but many metabolites are unique to certain lineages. The network of specialized metabolism continuously diversified, simplified or reshaped during the evolution of streptophytes. Many routes of pan-plant specialized metabolism are involved in plant defence. Biotic interactions are recalled as major drivers of lineage-specific metabolomic diversification. However, the consequences of this diversity of specialized metabolism in the context of plant terrestrialization and land plant diversification into the major lineages of bryophytes, lycophytes, ferns, gymnosperms and angiosperms remain only little explored. Overall, this hampers conclusions on the evolutionary scenarios that shaped specialized metabolism. Recent efforts have brought forth new streptophyte model systems, an increase in genetically accessible species from distinct major plant lineages, and new functional data from a diversity of land plants on specialized metabolic pathways. In this review, we will integrate the recent data on the evolution of the plant immune system with the molecular data of specialized metabolism and its recognition. Based on this we will provide a contextual framework of the pan-plant specialized metabolism, the evolutionary aspects that shape it and the impact on adaptation to the terrestrial environment.

This article is part of the theme issue ‘The evolution of plant metabolism’.

## 1. Introduction

**Figure 1.**
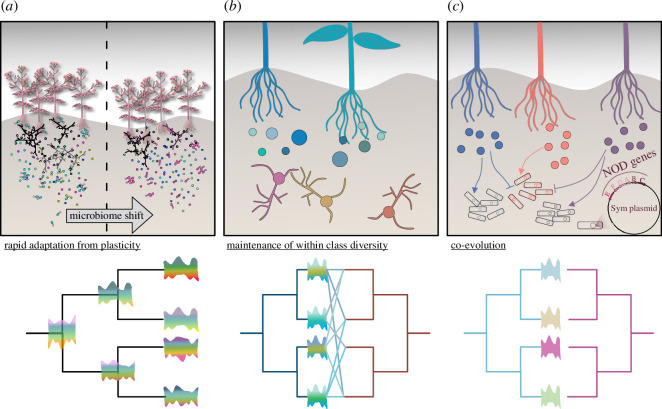
A schematic overview of context-dependent evolutionary trajectories for metabolome diversity in the green lineage. (*a*) Rapid adaptation from plasticity: phenotypic plasticity in the metabolome is maintained allowing for rapid adaptation of plant populations. Over evolutionary time, profiles will diverge between species owing to a mixture of environmental adaptation and drift, while diversity in itself is maintained. (*b*) Maintenance of within-class diversity: diversity within a class of metabolic compounds is maintained owing to the multiple interactions a plant has and the differential role of each compound. In this scenario, subsets of the same metabolic profile are required for the functional establishment of multiple symbiotic interactions, or protection against several pathogens/parasites. The profiles need to be adaptable depending on the microbiome context the host plant is confronted with, while at the same time, is required for some specificity in an interaction. In this scenario weak co-evolution may take place for certain subsets of compounds, while diversity is maintained to some degree. (*c*) Co-evolution: the metabolic profile of lineages diverges because a certain compound is required to maintain a specific interaction (attractor or specific toxin). Therefore, the compound profile is subjected to strong co-evolutionary changes to maintain a balanced interaction.

The specialized metabolism of the green lineage is at the core of plant–microbial interactions. This is independent of the type of interactions plants are faced with. Plants communicate with their associated microbiome via specialized chemicals, whether this regards attraction, repression, recognition or response to beneficial, commensal or pathogenic interactions. The evolutionary history of the biosynthesis pathways of these metabolites is arguably different for each of them. Metabolites involved in, for example, the attraction of beneficial microbes can be specific so that only certain microbial lineages recognize them [1,2] and are shaped by co-evolution between the partners (figure 1). On the other hand, some metabolites that are involved in repression, so-called phytoalexins, are functioning via rather unspecific defence mechanisms with broad-spectrum inhibition of microbial growth. One example here is Camalexin (see glossary, table 1), which has broad-spectrum inhibition against fungal pathogens [3,4]. Having said this, it is not that these compounds are exempt from co-evolution between plants and their pathogens. Many pathogens may have evolved detoxification mechanisms or inhibit the production of protecting compounds [5,6]. Moreover, lineage-specific anti-microbial compounds exist in abundance in land plants [7]. Thus, co-evolutionary forces in biotic interactions have been suggested as a main driver of the unprecedented metabolic diversity of the embryophytic specialized metabolism [8–11]. This is coherent with the observation that high lineage-specific variation is observed in many metabolic classes associated with defence (e.g. [12–14]).

**Table 1.** Glossary of specialized metabolites associated with plant-–microbe interactions salient to this work.

**Ascarosides:** ascarosides are glycosides consisting of short chain ω-hydroxylated fatty acids and the dideoxy sugar ascarylose produced by nematodes as pheromones. Ascarosides can be metabolized by Ar. thaliana, which results in the production of a nematode -repellent.
**Camalexin:** camalexin is an indole alkaloid, which has anti-microbial activities. It is specifically produced by Brassicaceae.
**Dinor−12-oxo-phytodienoic acid:** dn-OPDAs (dn-OPDA, dn-cis-OPDA, dn-iso-OPDA and Δ^4^-dn-OPDA) are bioactive oxylipins in the liverwort M. polymorpha and mount a jasmonate-based defence response. dn-OPDAs derive from the roughanic acid [16 : 3 (n−3) or (7Z,10Z,13Z)-hexadecatrienoic acid] pathway and both, dn-OPDA and dn-cis-OPDA can act in angiosperms as precursors for the phytohormone jasmonoyl-isoleucine.
**Ethylene:** ET is a gaseous phytohormone. Its synthesis and signalling are partially conserved across land plants and Zygnematophyceae.
**Flavonoids:** flavonoids and their derivatives derive from the phenylpropanoid pathway. They are involved in many biotic interactions including, but not limited to, pathogen defence and interactions with beneficial microbes, such as cyanobacteria and rhizobia.
**Jasmonic acid:** JA is an oxylipin. Its isoleucine conjugate is a phytohormone and derives either from roughanic acid (see dn-OPDA) or α-linolenic acid [18 : 3 (n−3) or (9Z,12Z,15Z)-octadecatrienoic acid].
**Lipochitooligosaccharides:** LCOs are based on a chitin (N-acetylglucosamine) backbone modified with functional groups, including long-chain fatty acids. LCOs are produced by bacteria and fungi and play a role in symbiotic communication between rhizobia and legumes as well as arbuscular and ectomycorrhizal fungi with their plant hosts.
**Phenylpropanoids:** phenylpropanoids are phenolic compounds that derive from phenylalanine or tyrosine and serve as the precursors for many so-called specialized metabolites, such as stilbenes, flavonoids and many more.
**Salicylic acid:** SA is a phenolic compound and phytohormone. It derives from the shikimate pathway either from isochorismate or phenylalanine. In Ar thaliana, the isochorismate-based route is the canonical and probably singularly used one. In other land plants, SA is synthesized from phenylalanine.
**Strigolactones:** SLs are isoprenoid phytohormones and derive from the 1-deoxy-d-xylulose 5-phosphate (DOXP) pathway. They are chemically based on a tricyclic lactone linked to a hydroxymethyl butanolide. Variability in SLs is mainly characterized by different residues on the A and B rings of the tricyclic lactone. SLs are involved in symbiotic interactions with, for example, AM fungi.
**Terpenoids:** Terpenoids are isoprenoids that derive either from the DOXP or mevalonate pathway. They include diterpenoid phytoalexins and carotenoids, but also several phytohormones, such as gibberellic acid, brassinosteroids, and ABA.

At the heart of integrating and responding correctly to plant–microbial interactions sit the phytohormones [15]. While their synthesis and responses to them are also consistently subjected to microbial manipulation, either by similar and/or mimicking molecules produced by microbes or effector-based manipulation of the biosynthesis or signalling pathways, these are widely conserved across major lineages of land plants [16]. This may be because they are part of bigger networks that are interwoven with each other and integrate all internal and external cues to regulate everything from development to responses to environmental cues. Nevertheless, phytohormones and their recognition and signalling pathways have very distinct trajectories during land plant evolution and the recent push forward in new model systems from under-represented land plant and algal lineages have allowed us first insights into the trajectory of these, but also other defence metabolites.

With the rise in new model systems, a central question that arises is how real the macroevolutionary diversity that we see, is in reality. While there is undoubtedly lineage-specificity [13], some of the macroevolutionary differences that we see may derive from the lack of representative data from a diversity outside the angiosperms [17]. Other diversity, however, may lie unexplored. An example of this is the phenylpropanoid pathway (PPP; see glossary, table 1). The PPP is the source of many compounds associated with defence, but several compounds derived from it are important for diverse aspects: for example flavonoids and derivatives (see glossary, table 1) are involved in diverse beneficial and detrimental biotic interactions or provide protection against abiotic stress. Lignin, which provides rigid cell walls and stability, allows upward growth. Here, alternative routes of the PPP towards lignin than those known from flowering plants were, for example, found in the lycophyte *Selaginella moellendorfii* [18–20]. Likewise, the genome of the aquatic lycophyte *Isoetes taiwanensis* showed evidence of the lycophyte route towards lignin with orthologs to the lycophyte CAFFEIC ACID/5-HYDROXYFERULIC ACID *O*-METHYLTRANSFERASE (COMT) and FERULATE 5-HYDROXYLASE (F5H), while missing the angiosperm COMT and F5H homologs [21]. While being broadly explored from an evolutionary point of view, the hypothesis that the PPP was an acquisition of land plants has been under investigation for years [22–29]. The acquisition of the entry enzyme PHENYLALANINE AMMONIA-LYASE (PAL) via horizontal gene transfer (HGT) into land plants was proposed to be the kick-off for the production of plant phenylpropanoids (PP; [25]). Yet, the streptophyte alga *Klebsormidium nitens* also possesses a PAL enzyme [27], which after extensive sampling of streptophyte algal lineages and recent phylotranscriptomics of the Klebsormidiophyceae suggests to be a second independent HGT into a terrestrial ancestor of the Klebsormidiophycean lineages *Interfilum* and *Klebsormidium* [30]. Despite the lack of PAL candidates in other streptophyte algal genomes, flavonoids have been detected in the streptophyte alga *Penium magaritaceum* and lignin-like compounds in, for example, *Nitella* and Coleochaetales [22,24,26,28]. Indeed, recent findings identified a PAL in *Chara braunii* [31], which clusters with HISTIDINE AMMONIA-LYASEs (HALs) in phylogenetic analyses [29]. Taken together, these data across the streptophytes suggest that this pathway is not a land plant innovation, but that it rather is modular and already integrated earlier into the green lineage. This is also supported by the high functional plasticity of enzymes involved in the synthesis of many specialized metabolic pathways. Here, a prime example is the CYP450 family [32]. CYP450 enzyme evolution is remarkably hard to study: For one, CYP450, enzymes show massive radiation with high lineage-specificity in the green lineage [33–35]. Second, functionally similar enzymes have been revealed as polyphyletic [18,34,36]. Additionally, these enzymes may show high substrate promiscuity when tested *in vitro* (e.g. [37]). They and other enzymes with similar evolutionary and functional characteristics, for example, UDP-DEPENDENT GLYCOSYLTRANSFERASEs, are involved in many of the pathways of specialized metabolism [38]. In evolutionary terms, this picture suggests that many pathways in specialized metabolism are mouldable by environmental shifts. As such, we may also underestimate the within-lineage possibilities of metabolite production.

Indeed, with the genomic investigation of a broader macroevolutionary diversity of land plants and algae, the core of many pathways appears to be present in some algal lineages. Here, either homologs of enzymes or pathways that have been said to be ‘land plant-specific’ are found in genomes of streptophyte algae or metabolites that derive from particular backbone structures, characteristic of land plants, have now been identified in algae. While the latter may also suggest parallel evolution of the biosynthetic pathway of a certain compound [7] it at its minimum requires the presence of a complete synthesis pathway of the backbone structure. These examples include the above-mentioned PPP pathway, for which by now most homologs and some PP derivatives across streptophytes (including the streptophyte algae) have been characterized, but also extend to other pathways. This may be true for phytohormones as well. Auxins including indole-3-acetic acid (IAA), for example, have been detected in several streptophyte algae [16]. This may *per se* not be surprising as many eukaryotic and prokaryotic lineages appear to synthesize auxins, yet no fully universal route of synthesis exists despite some common themes for IAA biosynthesis [39]. Now, several studies have investigated the presence of the plant pathway to IAA as well as the genetic makeup required for land plant responses and transport [40–42]. Also, other hormones, such as cytokinins and the terpenoid-derived hormone abscisic acid (ABA; see glossary, table 1) are produced in streptophyte algae [16]. Having said this, some variation, for example in recognition and downstream signalling exist within streptophytes [43–45].

Taken together, while many transitions during land plant evolution have recently been associated with the reshaping of metabolism [46], this begs the questions of whether (i) new inventions of land plants include any core backbone-producing pathways, and (ii) the increase in diversity in secondary metabolism during the evolution of land plants is true. For one, the latter still derives from a view of land plant evolution that was not yet aware of the deep dichotomy of land plants [47]. Second, it has to deal with a database considerably biased towards angiosperms. If indeed routes to many of the backbone structures of specialized metabolism are functionally conserved across the green lineage, this would suggest that the earliest land plants already had the toolkit for a majority of specialized metabolite core structures. They, like any other lineage, would then have diversified these sets upon their own need, and such diversification may have occurred via other modifications than typically known from angiosperms.

Nevertheless, different pathways are obviously under different selective pressures and underwent different evolutionary trajectories. In this review, we focus on the evolutionary history of specialized metabolism involved in the integration of biotic interactions. We investigate the evolutionary scenarios by which metabolism is driven to diversity within the framework of biotic interactions and discuss recent advances in functional characterization of defence-associated metabolites in new model land plants and algae. Here, we aim to infer the macroevolutionary trajectory of those pathways. Overall, we hypothesize that similar to a pan-genome, the green lineage has a pan-metabolome. This consists of a core that produces a certain range of backbone structures present to all representatives of the green lineage. Different evolutionary forces drive and maintain diversity in derivatives derived from the peripheral routes.

## 2. Evolutionary drivers of metabolic network diversity and their possible role in rapid adaptation of plants

Plants and their microbial partners are dependent on their communication. To this end, co-evolution of plants with their microbes is an important driver that shapes the evolutionary histories of all partners [48–51]. The effect that symbiotic interactions can have, is nicely illustrated in those symbioses that have resulted in organelles. For example, the chloroplast serves as a major hub for the synthesis of part of the specialized metabolism of plants and is essential for orchestrating metabolomic responses to biotic cues [52]. With regard to less-intimate associations, a paradox is how plants can match the evolutionary speed of their microbial associates [53]: Microbes have the ability to rapidly adapt to changes in their hosts and/or new (host) environments, owing to their short generation times. Yet despite their differences in generation time, plants are able to resist pathogens and uphold the communication to their beneficial symbionts. In addition to shorter generation time, HGT between microbial organisms increases the possibility for rapid adaptations that plants may need to be able to react to. Here, HGT has been suggested as a source for cheating in rhizobial symbioses with legumes [54].

Indeed, a novel environmental setting can induce phenotypic plasticity [55]; particularly with regard to metabolic responses, where the substrate environment is essential for the outcome, it is not complicated to envision that this can act as a pool for metabolic plasticity with which plants could encounter their pathogens or have a differential metabolomic recruitment based on the environment. Indeed, in snapdragon, wounding-associated emission of phenylacetaldehyde is dependent on a shift of phenylalanine synthesis from the plastidial to a cytosolic route and this shift is based on the expression of an isoform encoding for AROGENATE DEHYDRATASE 3, which lacks its plastid localization signal [56]. Moreover, not only is the rhizosphere microbiome shaped by the metabolic exudates from roots, but also the microbiome itself shapes the chemotype of the plant root metabolome [57–59]. Another example is the specific recruitment of leaf metabolomes towards the root colonization of an arbuscular mycorrhizal (AM) fungus [60]: Upon root inoculation, angiosperm plants significantly change their leaf metabolome in response to AM colonization. These changes are host-specific, in line with general species-specific leaf chemotypes; only a minimal phylogenetic conservation of chemotype modulation was detected. The majority was explained by differences between species and a host-specific modulation of the metabolome in response to the same AM species.

On a macroevolutionary scale, such plasticity as well as noisy metabolic background and enzyme promiscuity are a perfect set-up to have led to a different specialized metabolism [7,61–63]. One of the major drivers underlying the mechanism to achieve specificity but retain metabolic diversity is found in gene duplication from deep splits to recent lineage-specific radiations in gene families, which is followed by sub- and neo-functionalization [7,61,62]. The evolutionary history of the resulting diversity can be explained by a multitude of evolutionary scenarios that reach from drift to constructive neutral evolution to a point where co-evolution of plants with their microbial partners can act as an essential driver of metabolic divergence ([63]; figure 1). Co-evolutionary scenarios for driving the evolution of metabolism may be more complex than the well-explored resistance-gene effector-based co-evolutionary scenarios, in part, because one compound can have different effects on different organisms; and for another reason, because the metabolomic cross-talk has to be regarded from a holobiont perspective with multiple metabolic in- and outputs [51,64]. Yet, they exist. One being, for example, the co-ordinated symbioses between legumes and rhizobia that rely on distinct production of flavonoids and lipochitooligosaccharides (LCOs; see glossary, table 1; [65,66]). Second, receptors for strigolactones (SLs; see glossary, table 1) in parasitic plants are suggested to have expanded rapidly and evolved distinct sensitivity or affinity possibly to allow parasitic plants to sense a diversity of SLs from different hosts [67]. Another example is that of ascaroside pheromones (see glossary, table 1); the nematode-derived compounds can be metabolized by the plant to become a toxin for nematodes themselves [68].

In the end, specialized metabolism, however, has to remain plastic and cannot be selected towards only a few functional metabolites. That this is true is exemplified by the fact that not only one stimulant has a function, but rather that a mixture of metabolites of the same or different classes are produced at a similar time. For example, considering the ample literature on isolation and germination properties of SLs (which have several roles, e.g. in symbioses and as phytohormones for the hosts themselves) it appears that different SLs isolated from the same plant species are able to induce germination of different parasitic plants (for an overview see [67]). This function is independent of whether or not the plant that synthesizes them is an actual host species [69]. Similarly, in AM fungi, several different natural SLs induce spore germination and hyphal branching of the AM fungus *Gigaspora margarita* [70]. From a co-evolutionary point of view, this would suggest that there is some, but possibly not strong, selection towards a specific compound, but rather for the production of a number of certain classes of metabolites. When it comes to nitrogen-fixing symbioses of rhizobia, attractant host flavonoids act as specific inducers of *nodD* genes, where different flavonoid compositions have different effects on induction success to a degree where they can determine host-specificity [1]. Further host-specificity is then gained by the production of species-specific LCOs in the bacteria which are required for nodulation [71,72]. In these cases, strong co-evolutionary forces may act on metabolic recognition. Cyanobacterial nitrogen-fixing symbioses on the other hand appear specific on the plant side and flexible on the receiving bacterial side. In the cycad *Cycas revoluta,* 1-palmitoyl-2-linoleoyl-*sn*-glycerol was identified as an inducer of motile structures of the cyanobacteria [73]. Likewise, the hornwort *Anthoceros* produces a not-further determined metabolite, which is heat-labile, that can induce the motile structures [74]. In the angiosperm *Gunnera*, current data suggest it to be a peptide [75]. By contrast, attraction to a plant may be facilitated by compounds that are non-specific to hosts and it possibly relies on sugar metabolism [76,77]. This suggests that the establishment of cyanobacterial symbioses relies in part on a mixture of co-option of existing molecules and conserved primary metabolites. As such, this already suggests that there is no generic evolutionary scenario by which the diversity of metabolism associated with biotic interaction evolved. It appears rather context-dependent; AM fungi colonize approximately 70%–90% of all land plants [78], and these interactions probably date back until the time of terrestrialization [50,79–82]. By contrast, rhizobia are restricted to legumes. Cyanobacterial symbioses are lineage-specific but appear across diverse plant lineages [83]. The only exception is the permanent association of *Azolla* with its cyanobiont, which shows strong signs of co-evolution [48]. We would thus assume that compounds, which maintain a diversity of interactions and at the same time may also be exploited, are driven towards diversity, whereas compounds that maintain specific interactions are more likely to be subjected to selective sweeps. Compounds that are important for the stability of responses on the other hand may have more evolutionary constraints on their biosynthesis or have multiple routes for biosynthesis, which ensures reliable production.

What all of these compounds have in common is that they not only function in biotic interactions but are also part of other processes. As such, it is logical to assume that otherwise implemented compounds have been co-opted over long evolutionary timescales to become integral in biotic interactions. This may be even because they have been hijacked by pathogens, as could be envisioned for phytohormones that constitute hubs to abiotic, biotic and developmental responses; or, alternatively, because these metabolites are side-products of the degradation of other metabolites that are used by pathogens and symbionts: either from pathogens/symbionts themselves, as is observed with ascarosides, or vice versa, are microbe-degraded plant molecules, as would be the case, for example, for damage-associated molecular patterns. This would explain why diverse classes of metabolites that stem from the same core biosynthetic routes have similar or even overlapping functions in plant–microbe interactions. Here, the strength of selection towards the specificity of certain communication signals seems to depend on the intricacy of a particular interaction: where, for example, as a first layer in pathogen defence a cocktail of diverse phytotoxins is used, and intricate symbiotic relationships select for specific recognition of certain compounds. Additional plasticity in a metabolic cocktail is inert to plants, possibly owing, but not limited, to the contextual promiscuity of the enzymes involved in the biosynthesis. Such promiscuity can allow for rapid adaptation to either (i) new environments with a shift in metabolization capacity of the microbial community, as well as (ii) rapid upcoming of pathogenic isolates with the capacity to metabolize a previously successful toxin. As such, the co-evolutionary arms race between microbes and their hosts would lead to maintenance of promiscuity and plasticity.

## 3. The evolutionary histories of different phytohormone-based defence systems

We have discussed how biotic interactions may play a role in allowing for both within-species metabolic diversity and high lineage-specificity with distinct roles for certain compounds. This routes in the flexibility of evolved enzymes, domain co-option and neo-functionalization, where different evolutionary forces act upon the context-dependent promiscuity of the enzymes, depending on the resulting metabolites’ activity. However, diversity not only exists on the level of metabolites, but consequently also in their recognition and downstream signalling cascades. This again is dependent on the context we are considering. Metabolite classes where different derivatives act rather similarly may require more promiscuous receptors, whereas metabolites with highly specific functions either require highly specific recognition and/or specific signal integration. For example, the recognition of LCOs as well as the differentiation of those from chitin requires recognition by unique combinations of LysM receptor kinases [84]. Yet, even for some phytohormones with broad function, and involved in defence, that seem to have a conserved occurrence across land plants, recent data suggest that there is more diversity on the macroevolutionary scale than first meets the eye.

In the following paragraphs, we will focus on the different evolutionary paths of defence-integrated metabolites, starting with phytohormones. The most prominent examples are the two phytohormones jasmonoyl-isoleucine (JA-Ile) and salicylic acid (SA; see glossary, table 1). Both jasmonates and SA appear to be recruited in some manner for defence responses across land plants [85,86], however, neither their exact functional integration in the defence system nor their signalling appears to be conserved.

Based on several studies in angiosperms, JA-Ile was established as one of the central players in defence against insects and necrotrophic pathogens (those that kill and thrive on their dying hosts) [87]. Gene expression profiles even suggest that also gymnosperms and bryophytes use this system for defence [88,89], and genomic data confirmed that homologs for genes that encode proteins, which synthesize and recognize both hormones appeared to be present (e.g. [90–92]). Yet, actual measurements in the moss *Physcomitrium patens* questioned the existence of JA-Ile in the organism, although precursors are present [93]. Indeed, a systematic study showed that JA itself was indeed not present in many mosses [94]. Other land plant lineages showed no, low levels or patchy, lineage-specific synthesis of the hormone [94], leaving a conundrum as to when jasmonate-based defence first appeared in land plant evolution. Several studies followed these, testing the candidate signalling cascade in the liverwort *Marchantia polymorpha* revealing that perception of precursors of JA-Ile and the downstream signalling is majorly conserved in bryophytes and angiosperms and controls similar processes [95–97]. Indeed, the ligand in the bryophyte seems to be the jasmonate precursor *dinor*-12-oxo-phytodienoic acid (*dn-*OPDA; see glossary, table 1). The mutation that changed the specificity from *dn*-OPDA to JA-Ile in the receptor CORONATINE INSENSITIVE 1 (COI1) first occurred in the ancestor of vascular plants [95]. What drove this mutation is as of yet unclear. What the data however suggest is that it coincides with differences in jasmonate synthesis: phylodiverse approaches for phytohormone characterization across the green lineage with a focus on (i) diversity in bryophytes and lycophytes [98], and (ii) a broader diversity of streptophyte algae [16], particularly within the Zygnematophyceae, which form the sister group to land plants [99], have been carried out. Overall, their data suggest that the JA-Ile precursor, JA, is predominantly produced in vascular plants and non-vascular lineages only with varying occurrences (and if so, often in very low amounts). In the diversity of streptophyte algae, Schmidt *et al*. [16] report JA only in representatives of the new order Klebsormidiales [30] and *Choleochaete*—and in the Klebsormidiales only in presumably stressed, stationary cultures. While the data by Chini *et al*. [98] suggest *dn-*OPDA production throughout streptophytes, a focus on the algae suggests a limitation of *dn-*OPDA production to some groups [16]. Despite the scattered occurrences of jasmonates in streptophyte algae, *K. nitens* may use a COI1-independent pathway for *dn-*OPDA recognition to respond to temperature stress, the mechanism of which is found in the liverwort *M. polymorpha* as well [100]. Whether also the mechanisms revolving around biotic defences are retained at least in some streptophyte algal lineages awaits the availability of a patho-system for this group.

Further, which jasmonate acts as a bioactive form is another matter. In *Arabidopsis thaliana,* the active compound is JA-Ile [101,102], yet methyljasmonate (MeJA) can be converted to JA-Ile [103]. However, differences in responses to these occur in the green lineage. In *Picea abies*, application of MeJA induces defence-associated gene expression and increased resistance to an insect and fungus [104,105], yet in the water fern *Azolla filiculoides* application of MeJA has only small effects on defence marker gene expression [106]. However, a recent study on an insect-derived toxin on *Azolla* further suggests—based on transcriptomic data—that *Azolla* probably has an active jasmonate metabolism [107]. In *M. polymorpha*, not only *dn-*OPDA but also a newly discovered COI1 ligand Δ^4^-*dn*-OPDA are bioactive and have an additive effect on jasmonate-dependent regulation of defence reponses [108].

In angiosperms, JA-Ile acts in concert with the gaseous phytohormone ethylene (ET; see glossary, table 1) [87]. ET is produced even in the Zygnematophyceaen alga *Spyrogyra pratensis* where it is also bioactive and the signalling cascade candidates of *S. pratensis* can in part functionally complement the homologs in *Ar. thaliana* [109]. Whether it is consistently functioning and integrated in defence responses even in the closest algal relatives of land plants remains a matter of investigation. Yet, its synthesis is conserved throughout streptophytes [110], with the exception of underwater angiosperms, such as seagrasses [111], or the aquatic fern *Az. filiculoides* [48]. Furthermore, ET signalling is consistently co-expressed with modules describing biotic interaction in *Zygnema* [110], suggesting that it might also here interplay with defence signalling.

SA has been described to be a defence signal mediating the response against biotrophic pathogens (those that cherish a living host) in angiosperms [87]. Yet, already in *Pi. abies,* SA-associated genes have been induced upon infection with the necrotrophic pathogen *Heterobasidion annosum* [88,104]. Similarly, SA accumulates upon infection of the moss *Ph. patens* with the necrotrophic pathogen *Botrytis cinerea* [112]. Yet, SA also accumulates upon infection of *M. polymorpha* with the biotrophic bacterial pathogen *Pseudomonas syringae* pv. *tomato* [113], and in *Az. filiculoides*, it is interlinked with its unique permanent symbiosis [106,114]. The link of SA to symbiosis has also been observed in other symbiotic systems during early proliferation and has been suggested as a mechanism to restrict overgrowth of the symbionts [115]. Whereas in *Az. filiculoides*, it appears to be involved in a rather more intricate manner [106,114]. Overall, this suggests that SA has an omnipresent role in regulating biotic interactions.

SA biosynthesis is described to derive either from direct cleavage of isochorismate or from phenylalanine via synthesis of benzoic acid [116]. The latter route does not contribute much to the overall SA levels in *Ar. thaliana* but appears more prominent in other angiosperms [117–119]. A recent study suggests that also *Chlamydomonas reinhardtii* probably employs a route via phenylalanine and benzoic acid [120], despite that isochorismate synthase (ICS) is conserved in the green lineage pointing to its function in naphtho- and anthraquinone biosynthesis [114,121]. The only exception here was the water fern *Az. filiculoides* that retains in its nuclear genome only genes coding for enzymes for those routes that synthesize SA via benzoic acid [114]. Additionally, a domain fusion of ICS with MenC/MenD domain(s) was observed in both studies for some algae and non-vascular plants [114,120] and highlighted as a possible reason for a PAL-initiated synthesis pathway in *C. reinhardtii* [120]. At least already three routes via benzoic acid have been thought of, a fully cytosolic one and versions that include peroxisome and/or mitochondria [116]. In different angiosperms, the function of enzymes from the different routes has been characterized [116]. Regarding the ICS pathway, the phylogenetic distribution of some of the enzymes involved already tells us that there will be variation within the angiosperms. Rekhter *et al*. [122] and Torrens-Spence *et al*. [123] have discovered that isochorismate in the angiosperm *Ar. thaliana* is cleaved by fusing to glutamate before it is cleaved into SA. The first step requires the GRETCHEN HAGEN 3 enzyme avrPphB SUSCEPTIBLE3, whereas the latter step was suggested to be either catalysed via ENHANCED PSEUDOMONAS SUSCEPTIBILTY 1 (EPS1) or spontaneous decay in *Ar. thaliana* [122,123]. EPS1 derives from an *Ar. thaliana*-specific duplication, which is co-orthologous to closely related sequences in other Brassicaceae [29,123], however, less closely related, homologous sequences from non-Brassicaceae species exist. Overall, this may suggest that other angiosperms rely on spontaneous conversion. Despite the lack of phylogenetic resolution, these first data already suggest that the two possibilities of SA biosynthesis are largely evolutionary conserved. Yet, the preference for the route taken is a more complicated matter. Here, a general preference in algae and non-vascular plants towards benzoic acid was suggested, whereas in angiosperms, current data would either suggest high lineage-specificity or parallel use of both pathways (e.g. [124–126]).

A similar enigma, that only recently gained more insight, is the perception and signalling of SA in plants other than *Ar. thaliana*. Here, *M. polymorpha* has been functionally investigated. In angiosperms, the BTB/POZ domain containing oligomer of the NONEXPRESSOR OF PATHOGEN-RELATED 1 (NPR1) is a positive regulator of SA via the family of TGACG MOTIF-BINDING FACTOR (TGA) transcription factors [127,128]. Additionally, NPR3 and NPR4 serve as negative regulators of SA signalling [128]. *Marchantia polymorpha* encodes one NPR, which was partially capable of functionally complementing *npr1* mutants of *Ar. thaliana* [129]. Similarly, the NPR candidate of the moss *Ph. patens* is also capable of restoring *Atnpr1* phenotypes, but resistance to the oomycete *Hyaloperonospora arabidopsidis* was not restored to its full strength [130]. When the candidate *MpNPR1* gene was investigated in the liverwort *M. polymorpha,* it was observed that NPR1 function in thermal stress appears to be conserved between *Ar. thaliana* and *M. polymorpha,* whereas in regard to defence it behaved as a suppressor [129]. This is interesting because it mirrors what we have seen in jasmonate and ET signalling; abiotic stress integration is deeply conserved across the green lineage. However, variation at least in SA, seems to exist with regard to biotic interactions. It further remains unclear whether other BTB/POZ candidates can be recruited to the scene. The hornwort *Anthoceros,* for example, has no direct NPR homologs, yet it has BTB/POZ candidates that could serve the same role [92].

Next to the phytohormones, which operate at a nanomolar scale upstream of signalling cascades and are generally considered signalling molecules, other compounds are the end product of a pathway and have a function, for example, as attractant or toxin. These include, but are not limited to, PPs and their derivatives such as coumarins or flavonoids. Some routes may also produce both, signalling and protectant molecules, or molecules that operate in both aspects. Here, both the PP and the isoprenoid pathway towards terpenes are examples [116,131]. Both result in derivatives able to accumulate as phytoalexins, pigments involved in protection against abiotic stress or attractants and produce precursors for phytohormones. Does this suggest other macroevolutionary trends and a different evolutionary history for pathways or those parts of the pathways that are the source of a broad range of metabolites versus pathways (or parts of larger pathways) with less diverse output? In case of the PPP and terpene synthesis routes, homologous genes probably coding for the enzymes from the core pathways have been phylogenetically found across land plants, and to some degree also in streptophyte algae [17]. Yet, while the routes towards lignin appear more ancient, routes known from angiosperms to some derivatives, such as flavonoids, seem to have been recruited later in the evolution of land plants [132,133]. Having said this, lineages that lack parts of the angiosperm routes are capable of flavonoid synthesis. Here, two examples are the fern *Az. filiculoides* and the above-discussed case of the streptophyte alga *Pe. margaritaceum* [28,134]. This suggests that some lineages may have recruited other enzymes to the pathway. Moreover, when we consider the PPP and its road to lignin, we see that there are many lineage-specific radiations [29]. This together with the enzyme promiscuity in the pathway, which in itself has lineage-specific dimensions [37] agrees with the different metabolite preferences and diversity that each lineage has. Additionally, there are probably unexplored routes into the PPP, given that the canonical entry enzyme PAL is limited to land plants and the streptophyte algae from the Klebsormidiales [30]. In agreement, as described above, a phylogenetically assigned HAL from *Chara braunii* can function as a PAL [31]. In the PPP, one promiscuous enzyme is following another, allowing from a microevolutionary perspective much flexibility, less dependence and the possibility of alternative routes upon pathogen-interference. This in turn can lead to a drive towards the preference and expansion of certain peripheral routes in one lineage over the other, while at the same time retaining diversity. As such, the enzymes in these pathways may be evolutionary less constrainted than those in other pathways. Indeed, both anthocyanin biosynthesis and terpenoid biosynthesis pathways show relaxed constraints in downstream enzymes of the pathways [135,136]. Highly connected enzymes, however, show somewhat more constraints, probably owing to interconnectivity [136]. Yet, in carrot branching point enzymes CRISTO, LYCE and LYCB1 in carotenoid biosynthesis appear to be under balancing selection [137]. In these studies, the constraint has been correlated with position in the pathway, whether promiscuity of enzymes plays a role has not been investigated.

## 4. Conclusion

Plant–biotic interactions have been consistently suggested as a driver of plant metabolic diversity [8–11]. However, the actual metabolic diversity of the green lineage is far from explored and any assumptions of it are biased by the lack of non-angiosperm data. The recent rise in genomics has however suggested that (i) the core of secondary metabolite pathways associated with environmental responses, including defence, is largely present throughout streptophytes, (ii) that much radiation has occurred in some of the pathways, and (iii) that other pathways show unexpected diversity in their biosynthesis and/or recognition and signalling cascades. In some pathways, enzyme promiscuity is complicating the matter. In this review, we have discussed the evolutionary scenarios that could act on metabolite diversity and further in detail discussed recent advances in some examples of metabolic and signalling pathways. We highlight that the evolution of metabolism towards its diversity is probably a function of drift, constructive neutral evolution as well as selection. To what degree which of these forces apply is a matter of the metabolites’ function, as the recurring theme to be formulated in all those pathways is probably flexibility in how to come to a certain outcome.

As such, we propose that a pan-specialized metabolome of plants exists, which is defined by a core set of specialized metabolites that are modified to give rise to the extreme lineage-specific diversity that is observed in the extant representatives of the green lineage. In the course of the evolution of the green lineage, the core metabolites have been maintained, albeit not necessarily the routes towards them. Selection mainly acts on the diversity in the peripheral routes, which is underpinned by a few studies showing relaxed selective constraints towards the later, specialized parts of pathways.

## Data Availability

This article has no additional data.
